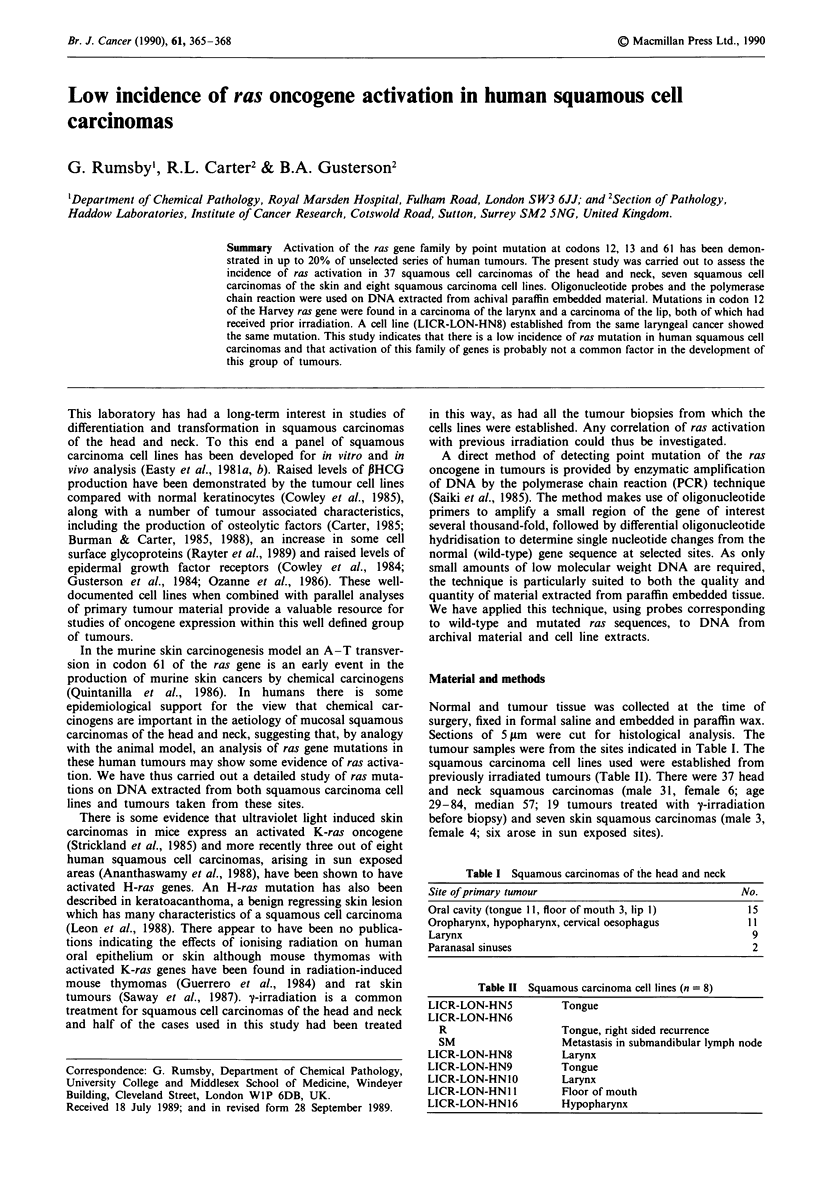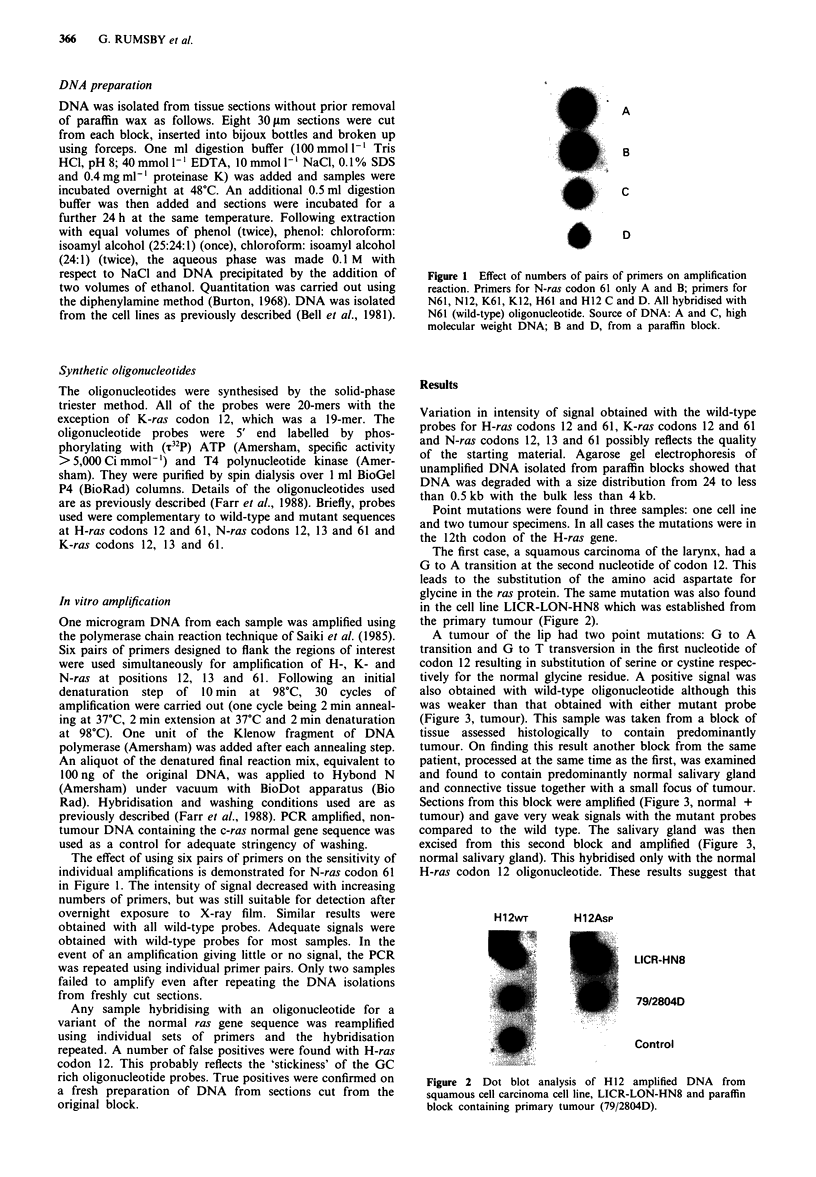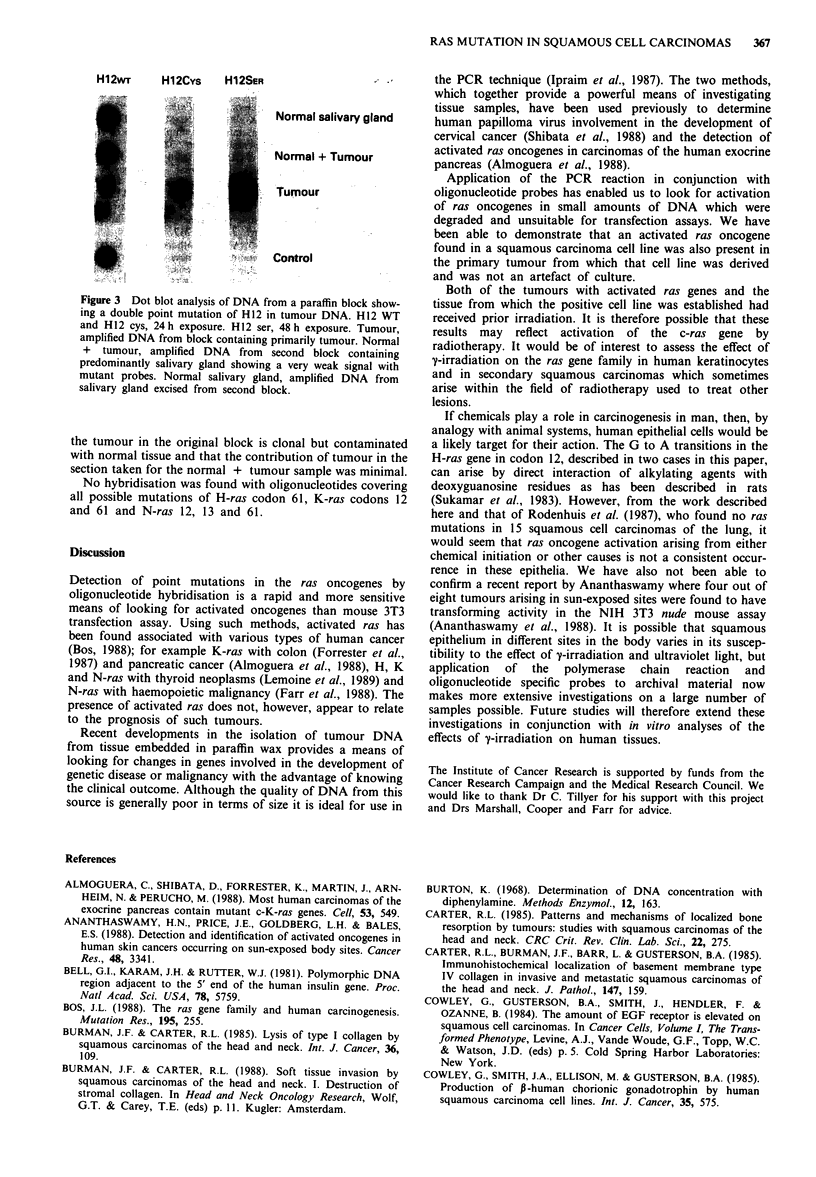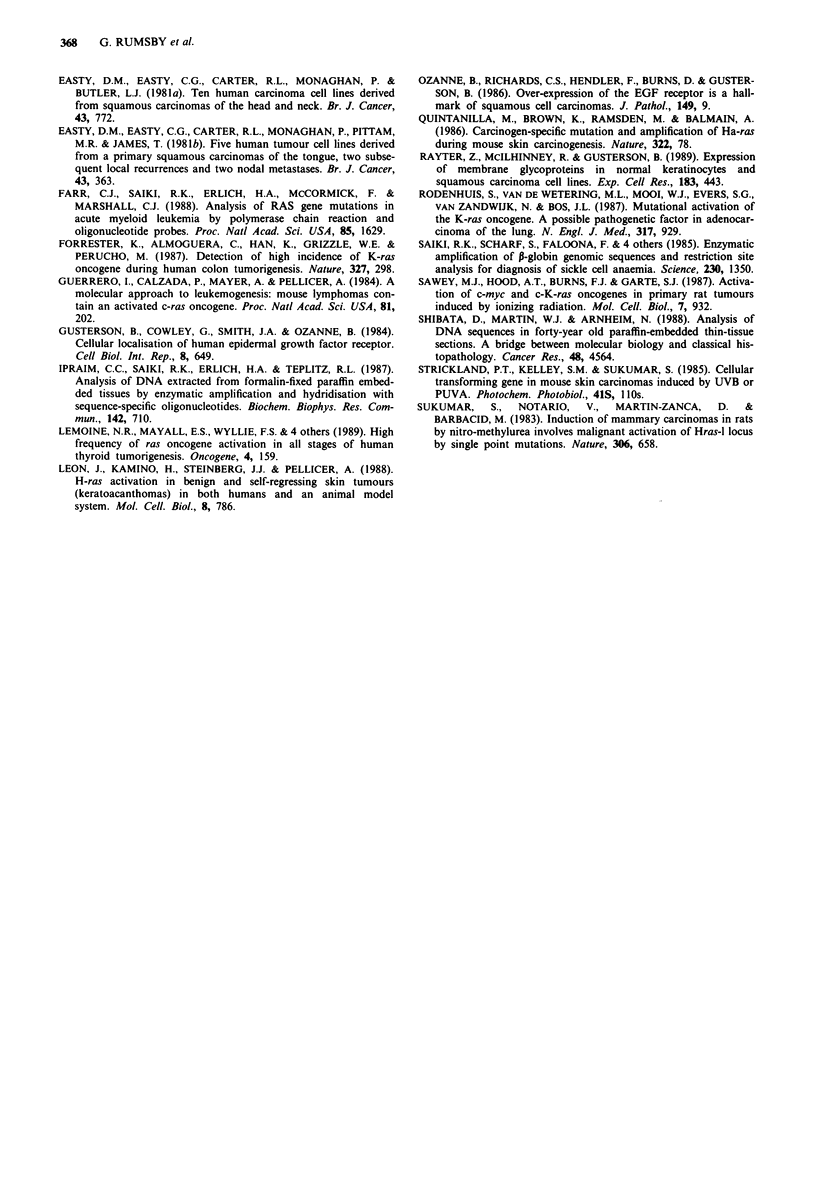# Low incidence of ras oncogene activation in human squamous cell carcinomas.

**DOI:** 10.1038/bjc.1990.80

**Published:** 1990-03

**Authors:** G. Rumsby, R. L. Carter, B. A. Gusterson

**Affiliations:** Department of Chemical Pathology, Royal Marsden Hospital, London, United Kingdom.

## Abstract

**Images:**


Br. J. Cancer (1990), 61, 365-368 © Macmillan Press Ltd., 1990

### Low incidence of *ras* oncogene activation in human squamous cell carcinomas

G. Rumsby[1], R.L. Carter[2] & B.A. Gusterson[2]

[1]*Department of Chemical Pathology, Royal Marsden Hospital, Fulham Road, London SW3 6JJ; and* [2]*Section of Pathology, Haddow Laboratories, Institute of Cancer Research, Cotswold Road, Sutton, Surrey SM2 5NG, United Kingdom.*

**Summary** Activation of the *ras* gene family by point mutation at codons 12, 13 and 61 has been demonstrated in up to 20% of unselected series of human tumours. The present study was carried out to assess the incidence of *ras* activation in 37 squamous cell carcinomas of the head and neck, seven squamous cell carcinomas of the skin and eight squamous carcinoma cell lines. Oligonucleotide probes and the polymerase chain reaction were used on DNA extracted from achival paraffin embedded material. Mutations in codon 12 of the Harvey *ras* gene were found in a carcinoma of the larynx and a carcinoma of the lip, both of which had received prior irradiation. A cell line (LICR-LON-HN8) established from the same laryngeal cancer showed the same mutation. This study indicates that there is a low incidence of *ras* mutation in human squamous cell carcinomas and that activation of this family of genes is probably not a common factor in the development of this group of tumours.

This laboratory has had a long-term interest in studies of differentiation and transformation in squamous carcinomas of the head and neck. To this end a panel of squamous carcinoma cell lines has been developed for *in vitro* and *in vivo* analysis (Easty *et al.*, 1981*a*, *b*). Raised levels of βHCG production have been demonstrated by the tumour cell lines compared with normal keratinocytes (Cowley *et al.*, 1985), along with a number of tumour associated characteristics, including the production of osteolytic factors (Carter, 1985; Burman & Carter, 1985, 1988), an increase in some cell surface glycoproteins (Rayter *et al.*, 1989) and raised levels of epidermal growth factor receptors (Cowley *et al.*, 1984; Gusterson *et al.*, 1984; Ozanne *et al.*, 1986). These well-documented cell lines when combined with parallel analyses of primary tumour material provide a valuable resource for studies of oncogene expression within this well defined group of tumours.

In the murine skin carcinogenesis model an A-T transversion in codon 61 of the *ras* gene is an early event in the production of murine skin cancers by chemical carcinogens (Quintanilla *et al.*, 1986). In humans there is some epidemiological support for the view that chemical carcinogens are important in the aetiology of mucosal squamous carcinomas of the head and neck, suggesting that, by analogy with the animal model, an analysis of *ras* gene mutations in these human tumours may show some evidence of *ras* activation. We have thus carried out a detailed study of *ras* mutations on DNA extracted from both squamous carcinoma cell lines and tumours taken from these sites.

There is some evidence that ultraviolet light induced skin carcinomas in mice express an activated K-*ras* oncogene (Strickland *et al.*, 1985) and more recently three out of eight human squamous cell carcinomas, arising in sun exposed areas (Ananthaswamy *et al.*, 1988), have been shown to have activated H-*ras* genes. An H-*ras* mutation has also been described in keratoacanthoma, a benign regressing skin lesion which has many characteristics of a squamous cell carcinoma (Leon *et al.*, 1988). There appear to have been no publications indicating the effects of ionising radiation on human oral epithelium or skin although mouse thymomas with activated K-*ras* genes have been found in radiation-induced mouse thymomas (Guerrero *et al.*, 1984) and rat skin tumours (Saway *et al.*, 1987). γ-irradiation is a common treatment for squamous cell carcinomas of the head and neck and half of the cases used in this study had been treated in this way, as had all the tumour biopsies from which the cells lines were established. Any correlation of *ras* activation with previous irradiation could thus be investigated.

A direct method of detecting point mutation of the *ras* oncogene in tumours is provided by enzymatic amplification of DNA by the polymerase chain reaction (PCR) technique (Saiki *et al.*, 1985). The method makes use of oligonucleotide primers to amplify a small region of the gene of interest several thousand-fold, followed by differential oligonucleotide hydridisation to determine single nucleotide changes from the normal (wild-type) gene sequence at selected sites. As only small amounts of low molecular weight DNA are required, the technique is particularly suited to both the quality and quantity of material extracted from paraffin embedded tissue. We have applied this technique, using probes corresponding to wild-type and mutated *ras* sequences, to DNA from archival material and cell line extracts.

### Material and methods

Normal and tumour tissue was collected at the time of surgery, fixed in formal saline and embedded in paraffin wax. Sections of 5 μm were cut for histological analysis. The tumour samples were from the sites indicated in Table I. The squamous carcinoma cell lines used were established from previously irradiated tumours (Table II). There were 37 head and neck squamous carcinomas (male 31, female 6; age 29-84, median 57; 19 tumours treated with γ-irradiation before biopsy) and seven skin squamous carcinomas (male 3, female 4; six arose in sun exposed sites).

**Table I** Squamous carcinomas of the head and neck

| *Site of primary tumour* | *No.* |
|---|---|
| Oral cavity (tongue 11, floor of mouth 3, lip 1) | 15 |
| Oropharynx, hypopharynx, cervical oesophagus | 11 |
| Larynx | 9 |
| Paranasal sinuses | 2 |

**Table II** Squamous carcinoma cell lines ($n = 8$)

| | |
|---|---|
| LICR-LON-HN5 | Tongue |
| LICR-LON-HN6 | |
| R | Tongue, right sided recurrence |
| SM | Metastasis in submandibular lymph node |
| LICR-LON-HN8 | Larynx |
| LICR-LON-HN9 | Tongue |
| LICR-LON-HN10 | Larynx |
| LICR-LON-HN11 | Floor of mouth |
| LICR-LON-HN16 | Hypopharynx |

Correspondence: G. Rumsby, Department of Chemical Pathology, University College and Middlesex School of Medicine, Windeyer Building, Cleveland Street, London W1P 6DB, UK.
Received 18 July 1989; and in revised form 28 September 1989.

*DNA preparation*

DNA was isolated from tissue sections without prior removal of paraffin wax as follows. Eight 30 μm sections were cut from each block, inserted into bijoux bottles and broken up using forceps. One ml digestion buffer (100 mmol $l^{-1}$ Tris HCl, pH 8; 40 mmol $l^{-1}$ EDTA, 10 mmol $l^{-1}$ NaCl, 0.1% SDS and 0.4 mg $ml^{-1}$ proteinase K) was added and samples were incubated overnight at 48°C. An additional 0.5 ml digestion buffer was then added and sections were incubated for a further 24 h at the same temperature. Following extraction with equal volumes of phenol (twice), phenol: chloroform: isoamyl alcohol (25:24:1) (once), chloroform: isoamyl alcohol (24:1) (twice), the aqueous phase was made 0.1 M with respect to NaCl and DNA precipitated by the addition of two volumes of ethanol. Quantitation was carried out using the diphenylamine method (Burton, 1968). DNA was isolated from the cell lines as previously described (Bell *et al.*, 1981).

*Synthetic oligonucleotides*

The oligonucleotides were synthesised by the solid-phase triester method. All of the probes were 20-mers with the exception of K-*ras* codon 12, which was a 19-mer. The oligonucleotide probes were 5′ end labelled by phosphorylating with $(\tau^{32}P)$ ATP (Amersham, specific activity $>$5,000 Ci $mmol^{-1}$) and T4 polynucleotide kinase (Amersham). They were purified by spin dialysis over 1 ml BioGel P4 (BioRad) columns. Details of the oligonucleotides used are as previously described (Farr *et al.*, 1988). Briefly, probes used were complementary to wild-type and mutant sequences at H-*ras* codons 12 and 61, N-*ras* codons 12, 13 and 61 and K-*ras* codons 12, 13 and 61.

*In vitro amplification*

One microgram DNA from each sample was amplified using the polymerase chain reaction technique of Saiki *et al.* (1985). Six pairs of primers designed to flank the regions of interest were used simultaneously for amplification of H-, K- and N-*ras* at positions 12, 13 and 61. Following an initial denaturation step of 10 min at 98°C, 30 cycles of amplification were carried out (one cycle being 2 min annealing at 37°C, 2 min extension at 37°C and 2 min denaturation at 98°C). One unit of the Klenow fragment of DNA polymerase (Amersham) was added after each annealing step. An aliquot of the denatured final reaction mix, equivalent to 100 ng of the original DNA, was applied to Hybond N (Amersham) under vacuum with BioDot apparatus (Bio Rad). Hybridisation and washing conditions used are as previously described (Farr *et al.*, 1988). PCR amplified, non-tumour DNA containing the c-*ras* normal gene sequence was used as a control for adequate stringency of washing.

The effect of using six pairs of primers on the sensitivity of individual amplifications is demonstrated for N-*ras* codon 61 in Figure 1. The intensity of signal decreased with increasing numbers of primers, but was still suitable for detection after overnight exposure to X-ray film. Similar results were obtained with all wild-type probes. Adequate signals were obtained with wild-type probes for most samples. In the event of an amplification giving little or no signal, the PCR was repeated using individual primer pairs. Only two samples failed to amplify even after repeating the DNA isolations from freshly cut sections.

Any sample hybridising with an oligonucleotide for a variant of the normal *ras* gene sequence was reamplified using individual sets of primers and the hybridisation repeated. A number of false positives were found with H-*ras* codon 12. This probably reflects the 'stickiness' of the GC rich oligonucleotide probes. True positives were confirmed on a fresh preparation of DNA from sections cut from the original block.

**Figure 1** Effect of numbers of pairs of primers on amplification reaction. Primers for N-*ras* codon 61 only A and B; primers for N61, N12, K61, K12, H61 and H12 C and D. All hybridised with N61 (wild-type) oligonucleotide. Source of DNA: A and C, high molecular weight DNA; B and D, from a paraffin block.

**Results**

Variation in intensity of signal obtained with the wild-type probes for H-*ras* codons 12 and 61, K-*ras* codons 12 and 61 and N-*ras* codons 12, 13 and 61 possibly reflects the quality of the starting material. Agarose gel electrophoresis of unamplified DNA isolated from paraffin blocks showed that DNA was degraded with a size distribution from 24 to less than 0.5 kb with the bulk less than 4 kb.

Point mutations were found in three samples: one cell ine and two tumour specimens. In all cases the mutations were in the 12th codon of the H-*ras* gene.

The first case, a squamous carcinoma of the larynx, had a G to A transition at the second nucleotide of codon 12. This leads to the substitution of the amino acid aspartate for glycine in the *ras* protein. The same mutation was also found in the cell line LICR-LON-HN8 which was established from the primary tumour (Figure 2).

A tumour of the lip had two point mutations: G to A transition and G to T transversion in the first nucleotide of codon 12 resulting in substitution of serine or cystine respectively for the normal glycine residue. A positive signal was also obtained with wild-type oligonucleotide although this was weaker than that obtained with either mutant probe (Figure 3, tumour). This sample was taken from a block of tissue assessed histologically to contain predominantly tumour. On finding this result another block from the same patient, processed at the same time as the first, was examined and found to contain predominantly normal salivary gland and connective tissue together with a small focus of tumour. Sections from this block were amplified (Figure 3, normal + tumour) and gave very weak signals with the mutant probes compared to the wild type. The salivary gland was then excised from this second block and amplified (Figure 3, normal salivary gland). This hybridised only with the normal H-*ras* codon 12 oligonucleotide. These results suggest that

**Figure 2** Dot blot analysis of H12 amplified DNA from squamous cell carcinoma cell line, LICR-LON-HN8 and paraffin block containing primary tumour (79/2804D).

**Figure 3** Dot blot analysis of DNA from a paraffin block showing a double point mutation of H12 in tumour DNA. H12 WT and H12 cys, 24 h exposure. H12 ser, 48 h exposure. Tumour, amplified DNA from block containing primarily tumour. Normal + tumour, amplified DNA from second block containing predominantly salivary gland showing a very weak signal with mutant probes. Normal salivary gland, amplified DNA from salivary gland excised from second block.

the tumour in the original block is clonal but contaminated with normal tissue and that the contribution of tumour in the section taken for the normal + tumour sample was minimal.

No hybridisation was found with oligonucleotides covering all possible mutations of H-*ras* codon 61, K-*ras* codons 12 and 61 and N-*ras* 12, 13 and 61.

### Discussion

Detection of point mutations in the *ras* oncogenes by oligonucleotide hybridisation is a rapid and more sensitive means of looking for activated oncogenes than mouse 3T3 transfection assay. Using such methods, activated *ras* has been found associated with various types of human cancer (Bos, 1988); for example K-*ras* with colon (Forrester *et al.*, 1987) and pancreatic cancer (Almoguera *et al.*, 1988), H, K and N-*ras* with thyroid neoplasms (Lemoine *et al.*, 1989) and N-*ras* with haemopoietic malignancy (Farr *et al.*, 1988). The presence of activated *ras* does not, however, appear to relate to the prognosis of such tumours.

Recent developments in the isolation of tumour DNA from tissue embedded in paraffin wax provides a means of looking for changes in genes involved in the development of genetic disease or malignancy with the advantage of knowing the clinical outcome. Although the quality of DNA from this source is generally poor in terms of size it is ideal for use in the PCR technique (Ipraim *et al.*, 1987). The two methods, which together provide a powerful means of investigating tissue samples, have been used previously to determine human papilloma virus involvement in the development of cervical cancer (Shibata *et al.*, 1988) and the detection of activated *ras* oncogenes in carcinomas of the human exocrine pancreas (Almoguera *et al.*, 1988).

Application of the PCR reaction in conjunction with oligonucleotide probes has enabled us to look for activation of *ras* oncogenes in small amounts of DNA which were degraded and unsuitable for transfection assays. We have been able to demonstrate that an activated *ras* oncogene found in a squamous carcinoma cell line was also present in the primary tumour from which that cell line was derived and was not an artefact of culture.

Both of the tumours with activated *ras* genes and the tissue from which the positive cell line was established had received prior irradiation. It is therefore possible that these results may reflect activation of the c-*ras* gene by radiotherapy. It would be of interest to assess the effect of γ-irradiation on the *ras* gene family in human keratinocytes and in secondary squamous carcinomas which sometimes arise within the field of radiotherapy used to treat other lesions.

If chemicals play a role in carcinogenesis in man, then, by analogy with animal systems, human epithelial cells would be a likely target for their action. The G to A transitions in the H-*ras* gene in codon 12, described in two cases in this paper, can arise by direct interaction of alkylating agents with deoxyguanosine residues as has been described in rats (Sukamar *et al.*, 1983). However, from the work described here and that of Rodenhuis *et al.* (1987), who found no *ras* mutations in 15 squamous cell carcinomas of the lung, it would seem that *ras* oncogene activation arising from either chemical initiation or other causes is not a consistent occurrence in these epithelia. We have also not been able to confirm a recent report by Ananthaswamy where four out of eight tumours arising in sun-exposed sites were found to have transforming activity in the NIH 3T3 *nude* mouse assay (Ananthaswamy *et al.*, 1988). It is possible that squamous epithelium in different sites in the body varies in its susceptibility to the effect of γ-irradiation and ultraviolet light, but application of the polymerase chain reaction and oligonucleotide specific probes to archival material now makes more extensive investigations on a large number of samples possible. Future studies will therefore extend these investigations in conjunction with *in vitro* analyses of the effects of γ-irradiation on human tissues.

The Institute of Cancer Research is supported by funds from the Cancer Research Campaign and the Medical Research Council. We would like to thank Dr C. Tillyer for his support with this project and Drs Marshall, Cooper and Farr for advice.

### References

ALMOGUERA, C., SHIBATA, D., FORRESTER, K., MARTIN, J., ARNHEIM, N. & PERUCHO, M. (1988). Most human carcinomas of the exocrine pancreas contain mutant c-K-*ras* genes. *Cell*, **53**, 549.

ANANTHASWAMY, H.N., PRICE, J.E., GOLDBERG, L.H. & BALES, E.S. (1988). Detection and identification of activated oncogenes in human skin cancers occurring on sun-exposed body sites. *Cancer Res.*, **48**, 3341.

BELL, G.I., KARAM, J.H. & RUTTER, W.J. (1981). Polymorphic DNA region adjacent to the 5′ end of the human insulin gene. *Proc. Natl Acad. Sci. USA*, **78**, 5759.

BOS, J.L. (1988). The *ras* gene family and human carcinogenesis. *Mutation Res.*, **195**, 255.

BURMAN, J.F. & CARTER, R.L. (1985). Lysis of type I collagen by squamous carcinomas of the head and neck. *Int. J. Cancer*, **36**, 109.

BURMAN, J.F. & CARTER, R.L. (1988). Soft tissue invasion by squamous carcinomas of the head and neck. I. Destruction of stromal collagen. In *Head and Neck Oncology Research*, Wolf, G.T. & Carey, T.E. (eds) p. 11. Kugler: Amsterdam.

BURTON, K. (1968). Determination of DNA concentration with diphenylamine. *Methods Enzymol.*, **12**, 163.

CARTER, R.L. (1985). Patterns and mechanisms of localized bone resorption by tumours: studies with squamous carcinomas of the head and neck. *CRC Crit. Rev. Clin. Lab. Sci.*, **22**, 275.

CARTER, R.L., BURMAN, J.F., BARR, L. & GUSTERSON, B.A. (1985). Immunohistochemical localization of basement membrane type IV collagen in invasive and metastatic squamous carcinomas of the head and neck. *J. Pathol.*, **147**, 159.

COWLEY, G., GUSTERSON, B.A., SMITH, J., HENDLER, F. & OZANNE, B. (1984). The amount of EGF receptor is elevated on squamous cell carcinomas. In *Cancer Cells, Volume I, The Transformed Phenotype*, Levine, A.J., Vande Woude, G.F., Topp, W.C. & Watson, J.D. (eds) p. 5. Cold Spring Harbor Laboratories: New York.

COWLEY, G., SMITH, J.A., ELLISON, M. & GUSTERSON, B.A. (1985). Production of β-human chorionic gonadotrophin by human squamous carcinoma cell lines. *Int. J. Cancer*, **35**, 575.

EASTY, D.M., EASTY, C.G., CARTER, R.L., MONAGHAN, P. & BUTLER, L.J. (1981a). Ten human carcinoma cell lines derived from squamous carcinomas of the head and neck. *Br. J. Cancer*, **43**, 772.

EASTY, D.M., EASTY, C.G., CARTER, R.L., MONAGHAN, P., PITTAM, M.R. & JAMES, T. (1981b). Five human tumour cell lines derived from a primary squamous carcinomas of the tongue, two subsequent local recurrences and two nodal metastases. *Br. J. Cancer*, **43**, 363.

FARR, C.J., SAIKI, R.K., ERLICH, H.A., MCCORMICK, F. & MARSHALL, C.J. (1988). Analysis of RAS gene mutations in acute myeloid leukemia by polymerase chain reaction and oligonucleotide probes. *Proc. Natl Acad. Sci. USA*, **85**, 1629.

FORRESTER, K., ALMOGUERA, C., HAN, K., GRIZZLE, W.E. & PERUCHO, M. (1987). Detection of high incidence of K-*ras* oncogene during human colon tumorigenesis. *Nature*, **327**, 298.

GUERRERO, I., CALZADA, P., MAYER, A. & PELLICER, A. (1984). A molecular approach to leukemogenesis: mouse lymphomas contain an activated c-*ras* oncogene. *Proc. Natl Acad. Sci. USA*, **81**, 202.

GUSTERSON, B., COWLEY, G., SMITH, J.A. & OZANNE, B. (1984). Cellular localisation of human epidermal growth factor receptor. *Cell Biol. Int. Rep.*, **8**, 649.

IPRAIM, C.C., SAIKI, R.K., ERLICH, H.A. & TEPLITZ, R.L. (1987). Analysis of DNA extracted from formalin-fixed paraffin embedded tissues by enzymatic amplification and hydridisation with sequence-specific oligonucleotides. *Biochem. Biophys. Res. Commun.*, **142**, 710.

LEMOINE, N.R., MAYALL, E.S., WYLLIE, F.S. & 4 others (1989). High frequency of *ras* oncogene activation in all stages of human thyroid tumorigenesis. *Oncogene*, **4**, 159.

LEON, J., KAMINO, H., STEINBERG, J.J. & PELLICER, A. (1988). H-*ras* activation in benign and self-regressing skin tumours (keratoacanthomas) in both humans and an animal model system. *Mol. Cell. Biol.*, **8**, 786.

OZANNE, B., RICHARDS, C.S., HENDLER, F., BURNS, D. & GUSTERSON, B. (1986). Over-expression of the EGF receptor is a hallmark of squamous cell carcinomas. *J. Pathol.*, **149**, 9.

QUINTANILLA, M., BROWN, K., RAMSDEN, M. & BALMAIN, A. (1986). Carcinogen-specific mutation and amplification of Ha-*ras* during mouse skin carcinogenesis. *Nature*, **322**, 78.

RAYTER, Z., MCILHINNEY, R. & GUSTERSON, B. (1989). Expression of membrane glycoproteins in normal keratinocytes and squamous carcinoma cell lines. *Exp. Cell Res.*, **183**, 443.

RODENHUIS, S., VAN DE WETERING, M.L., MOOI, W.J., EVERS, S.G., VAN ZANDWIJK, N. & BOS, J.L. (1987). Mutational activation of the K-*ras* oncogene. A possible pathogenetic factor in adenocarcinoma of the lung. *N. Engl. J. Med.*, **317**, 929.

SAIKI, R.K., SCHARF, S., FALOONA, F. & 4 others (1985). Enzymatic amplification of β-globin genomic sequences and restriction site analysis for diagnosis of sickle cell anaemia. *Science*, **230**, 1350.

SAWEY, M.J., HOOD, A.T., BURNS, F.J. & GARTE, S.J. (1987). Activation of c-*myc* and c-K-*ras* oncogenes in primary rat tumours induced by ionizing radiation. *Mol. Cell. Biol.*, **7**, 932.

SHIBATA, D., MARTIN, W.J. & ARNHEIM, N. (1988). Analysis of DNA sequences in forty-year old paraffin-embedded thin-tissue sections. A bridge between molecular biology and classical histopathology. *Cancer Res.*, **48**, 4564.

STRICKLAND, P.T., KELLEY, S.M. & SUKUMAR, S. (1985). Cellular transforming gene in mouse skin carcinomas induced by UVB or PUVA. *Photochem. Photobiol.*, **41S**, 110s.

SUKUMAR, S., NOTARIO, V., MARTIN-ZANCA, D. & BARBACID, M. (1983). Induction of mammary carcinomas in rats by nitro-methylurea involves malignant activation of H*ras*-l locus by single point mutations. *Nature*, **306**, 658.